# Experimental and theoretical structural/spectroscopical correlation of enterobactin and catecholamide

**DOI:** 10.1016/j.dib.2018.08.114

**Published:** 2018-08-29

**Authors:** M. Moreno, A. Zacarias, L. Velasquez, G. Gonzalez, M. Alegría-Arcos, F. Gonzalez-Nilo, E.K.U. Gross

**Affiliations:** a Max Planck Institute of Microstructure Physics, Weinberg 2, D06120 Halle, Germany and ETSF.; b University of the Basque Country, Barrio Sarriena, s/n, 48940 Leioa, Bizkaia, Spain; c Universidad Andres Bello, Facultad de Medicina, Center for Integrative Medicine and Innovative Science, Echaurren 183, Santiago, Chile; d Center for Development of Nanoscience and Nanotechnology, CEDENNA, Casilla 653, Santiago, Chile; e Universidad de Chile, Facultad de Ciencias, Departamento de Química, Laboratorio de Sintesis Inorganica y electroquímica, Las Palmeras 3425, Nuñoa, Santiago, Chile; f Universidad Andres Bello, Facultad de Ciencias Biologicas, Center for Bioinformatic and Integrative Biology, Av Republica 239, Santiago, Chile; g Centro Interdisciplinario de Neurociencias de Valparaíso (CINV), Facultad de Ciencias, Universidad de Valparaíso, Valparaíso, Chile; h Facultad Ciencias de la Salud, Universidad SEK, Chile, Fernando Manterola 0789, Providencia, Santiago

**Keywords:** Catecholate FeEnterobactin, FTIR, DFT, MD

## Abstract

Here we report the IR spectra of FeEnterobactin in catecholate conformations ([CatFeEB]^3−^) obtained by DFT calculations using PBE/QZVP and their correlation it with its experimental counterpart [SalH_3_FeEB]^0^. Fragments of FeEnterobactin and Enterobactin (H_6_EB) are elucidated from their MALDI-TOF mass spectrometry, and the dependence of the frontier orbitals (HOMO and LUMO) with the catecholamide dihedral angles of H_6_EB is reported. The frequency distribution of catecholamide dihedral angle of H_6_EB was carried-out using molecular dynamics (MD). The data presented enriches the understanding of [CatFeEB]^3^^−^ and H_6_EB frequency distribution and reactivity.

**Specifications table**

**Table:** 

Subject area	Chemistry and biology.
More specific subject area	Synthesis, Functionalization, and Characterization of FeEnterobactin and Enterobactin, IR spectra, catecholamide dihedral angles distribution and reactivity.
Type of data	Plots were done with Origin 6.0 (OriginLab, Northampton, MA). We used Gauss-View to visualize the frontier orbitals, density, electrostatic potentials and vibrational modes.
How data was acquired	DFT calculations using PBE exchange/correlation functionals and QZVP basis set were used to obtain the infrared spectra (IR) of [SalFeH_3_EB]^0^ and H_6_EB. Experimental IR were recorded on a Brucker IFS66v/S vacuum FTIR spectrometer with a Ge/KBr beam splitter and DTGS detector, and the MALDI-TOF MS spectra were acquired with an Ultraflex II TOF-TOF mass spectrometer (Bruker Daltonics) for both samples (more details in *Spectrochim. Acta A (2018) 198, 264–277*). To obtain the frequencies distribution of different dihedral angles values (Arm1, Arm2, and Arm3, see Fig. 1, Fig. 2, Fig. 3, Fig. 4, Fig. 5, Fig. 6, Fig. 7) from H_6_EB structures over a time lapse, we used the Desmond code [4] to perform the molecular dynamics (MD) simulations for the four structures of H_6_EB. Each structure was embedded into an explicit TIP3P 2water box. The NPT ensemble was employed with at 300 K and 1.01 bar of pressure and the OPLS-2005 Force Field 3were used 4. Before the MD simulations, the energy of each system was minimized and then, MD simulations were carried out for 5 ns. We used a VMD program [5] to calculate the dihedrals angles on the catecholamides for the H_6_EB structures during the MD trajectory. Plots were done with Origin 6.0 (OriginLab, Northampton, MA). All systems were simulated considering periodic boundary conditions (PBC).
Data format	Figs. in TIF format.
Experimental factors	Experimental IR were recorded at 50000 scans with 2 cm^−1^ resolution. The sample, [SalFeH_3_EB]^0^ were prepared using KRS-5 disc. fifty milligrams of [SalFeH_3_EB]^0^ and H_6_EB, separately, was dispersed in 100 µl of dichloromethane, then one drop was placed on a KRS-5 disc to dry. Solid [SalFeH_3_EB]^0^ was characterized. All solvents were of analytical purity. For the sample preparation of MALDI-TOF MS spectra, 0.5 mL of a saturated solution of a-cyano-4-hydroxycinnamic acid (HCCA) in acetone was deposited on the sample target. A 1 ml aliquot of the sample was injected into a small drop of water previously deposited on the matrix surface [1].
Experimental features	Infrared Spectra of [SalFeH_3_EB]^0^ was carried out on solid state at RT, instead, a liquid state is performed to capture MALDI-TOF MS spectra.
Data source location	Theory and Experimental II departments of the Max Planck Institute of Microstructure Physics, Halle/Germany. Universidad Andres Bello, Facultad de Ciencias Biologicas, Center for Bioinformatic and Integrative Biology (CBIB).
Data accessibility	Data described here are Supplementary information to the article entitled “IR and NMR Spectroscopic Correlation of Enterobactin by DFT” Spectrochimica Acta A (2018) [1].
Related research article	Major details about Enterobactin IR spectra can be found in “IR and NMR Spectroscopic Correlation of Enterobactin by DFT” Spectrochimica Acta A (2018) [1]
	The Functionalization and characterization of Enterobactin and Fe Enterobactin analogs as well as their affinity prediction with FepA-protein transmembrane using DFT, Molecular Dynamics and Docking will be reported elsewhere.

**Value of the data**

• The elucidation of ([CatFeEB]^3−^) IR spectra by DFT contrasted with experimental IR leads a greater understanding of the functional group motion which favors the explanation of their chemical modification.
• The determination of the frequency distribution of dihedral angles of H_6_EB structures using molecular dynamics (MD) allows to reveal the predominant structure and with this, its prevailing electronic properties; their reactivity parameters leads to predict synthesis of new materials.
• The visualization of atomic bond cleavage of FeEnterobactin and Enterobactin obtained by mass spectrometry permit determine the reactivity sites useful for the implementation of functionalization methodologies.

## 1 Data

**Fig. 1:**
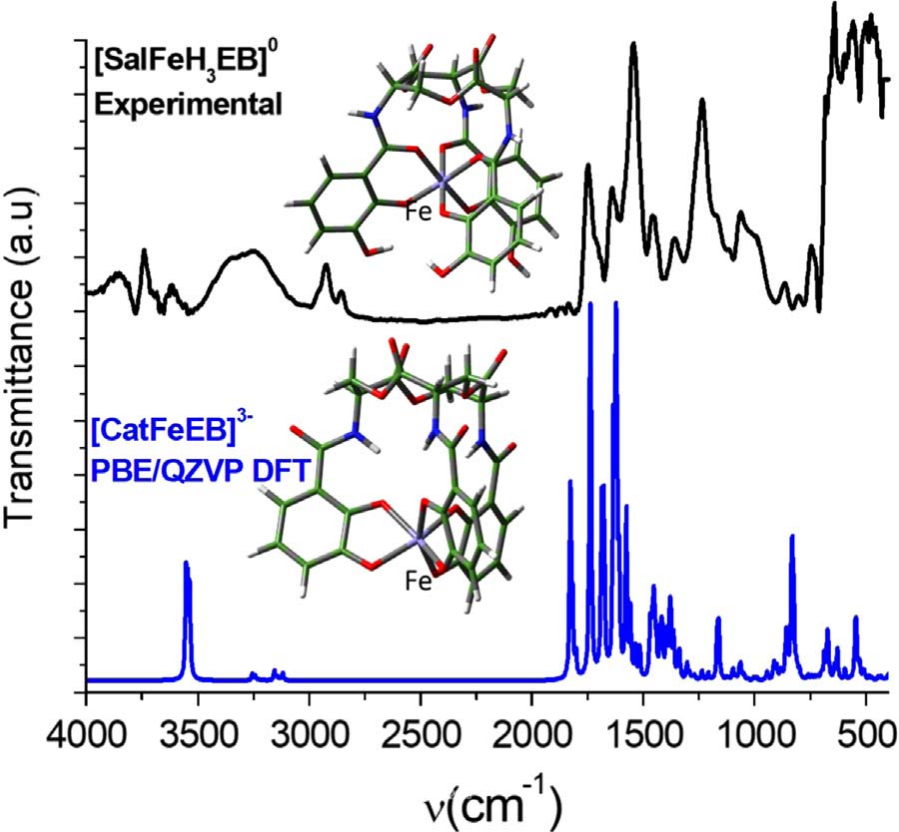
Calculated [CatFeEB]^3^^−^ IR spectra using PBE/QZVP method and Experimental [SalFeH_3_EB]^0^ in the range of 4000–450 cm^−^^1^. [CatFeEB]^3^^−^ corresponds to Fe linked at catechol groups, and [SalFeH_3_EB]^0^ to Fe at catecholamide groups respectively.

Fig. 1 shows the calculated catecholate FeEnterobactin ([CatFeEB]^3^^−^) contrasted with experimental [SalFeH_3_EB]^0^.

**Fig. 2:**
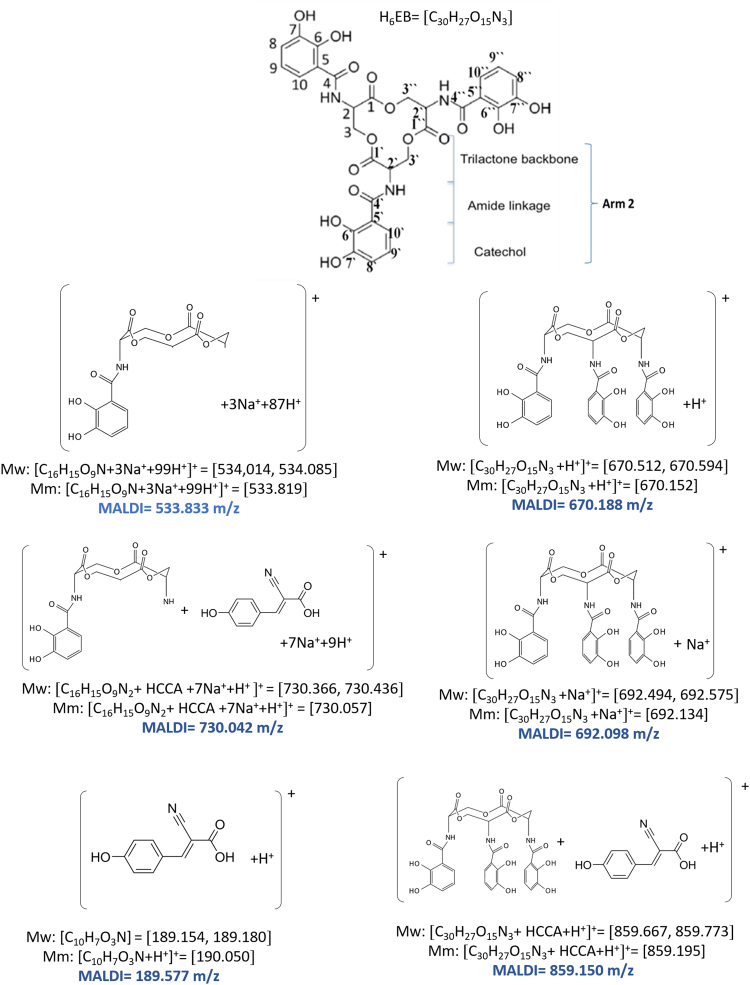
H_6_EB fragments based on MALDI-TOF MS spectra [1], calculated using minimum and maximum atomic weights (ma) from the IUPAC 2013 technical report [12], and Mm (monoisotopic mass) [16]. m_a_(H)= [1.00784, 1.00811]; m_a_(C)[12.0096, 12.0116], m_a_(N)[14.00643, 14.00728], m_a_(O)[15.99903, 15.99977] and m_a_(Na)[22.98977] were considered in the estimation of minimum and maximum molecular weights (Mw), and Mm was calculated using web tool provides by http://www.cheminfo.org. Being the average of the mass measurement error (or accuracy) of Δm:33.031 ppm (0.0033%) [16].

**Fig. 3:**
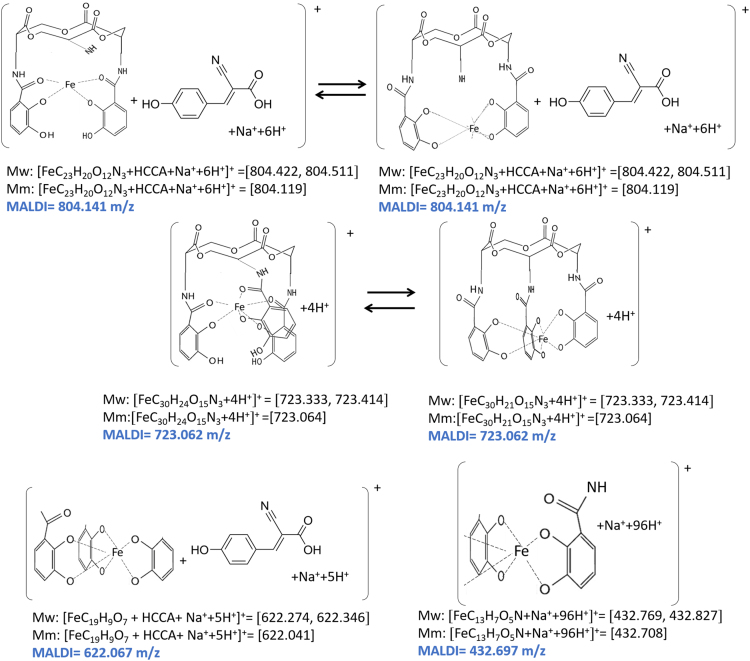
CatFeEnterobactin (CatFeEB) and SalFeH_3_Enterobactin (FeH_3_EB) fragments based on MALDI-TOF MS spectra [1], calculated using minimum and maximum atomic weights (ma) from the IUPAC 2013 technical report [12] and monoisotopic mass Mm [16]. m_a_(H)= [1.00784, 1.00811]; m_a_(C)[12.0096, 12.0116], m_a_(N)[14.00643, 14.00728], m_a_(O)[15.99903, 15.99977], m_a_(Na)[22.98977] and m_a_(Fe)[55.845] were considered in the estimation of minimum and maximum molecular weights (Mw), and Mm was calculated using web tool provides by http://www.cheminfo.org. Being the average of the mass measurement error (or accuracy) of Δm:11.625 ppm (0.0011%) [16].

**Fig. 4:**
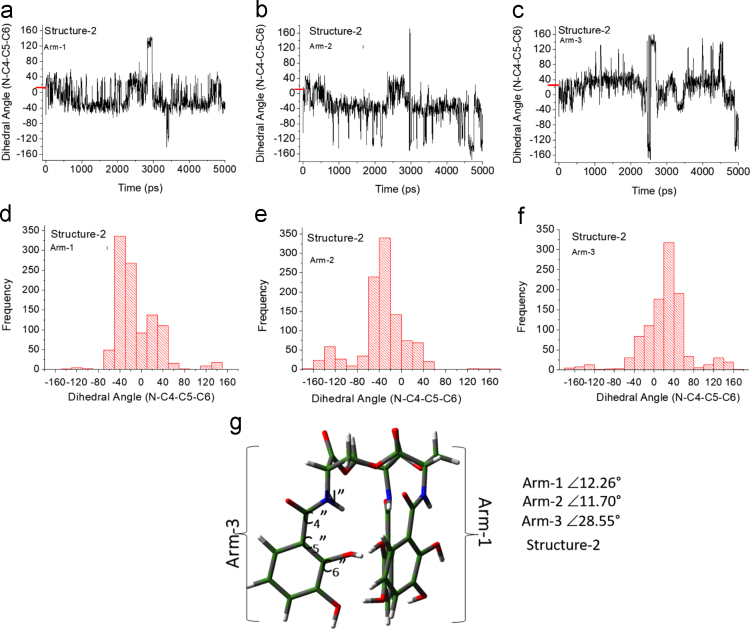
Dihedral angles of structure-2 arms (g) as a function of time (a-c) and frequency distribution (d-f).

**Fig. 5:**
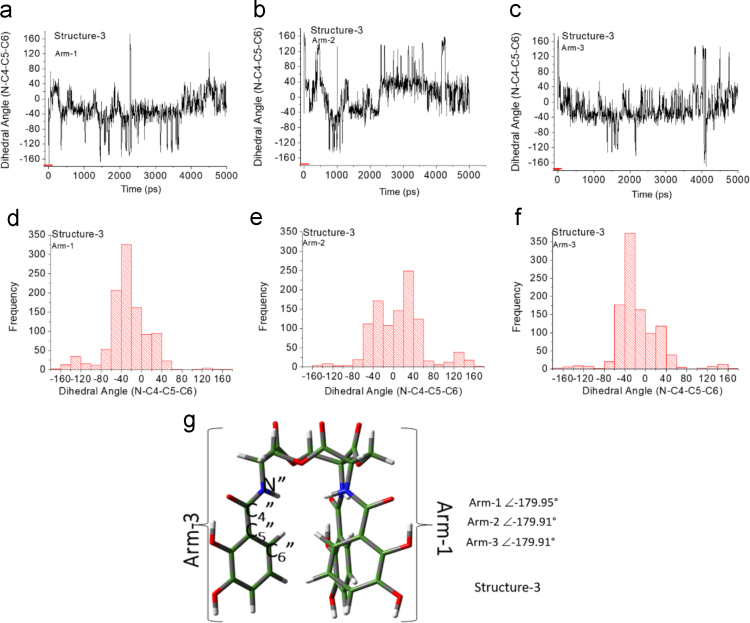
Dihedral angles of structure-3 arms (g) as a function of time (a-c) and frequency distribution (d-f).

**Fig. 6:**
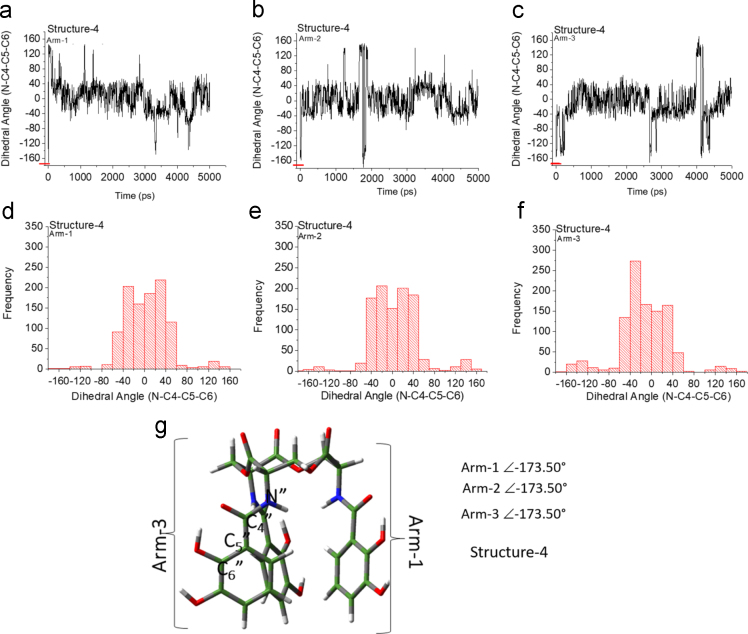
Dihedral angles of structure-4 arms (g) as a function of time (a-c) and frequency distribution (d-f).

**Fig. 7:**
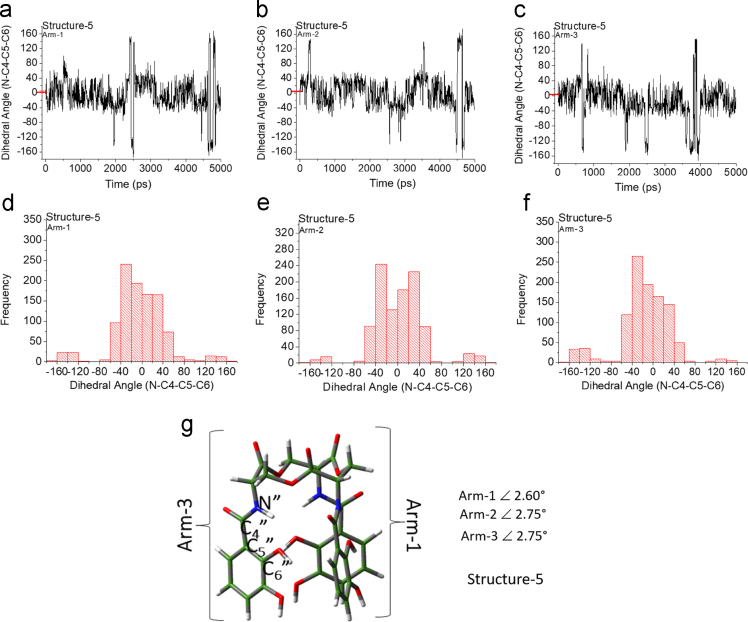
Dihedral angles of structure-5 arms (g) as a function of time (a-c) and frequency distribution (d-f).

**Fig. 8:**
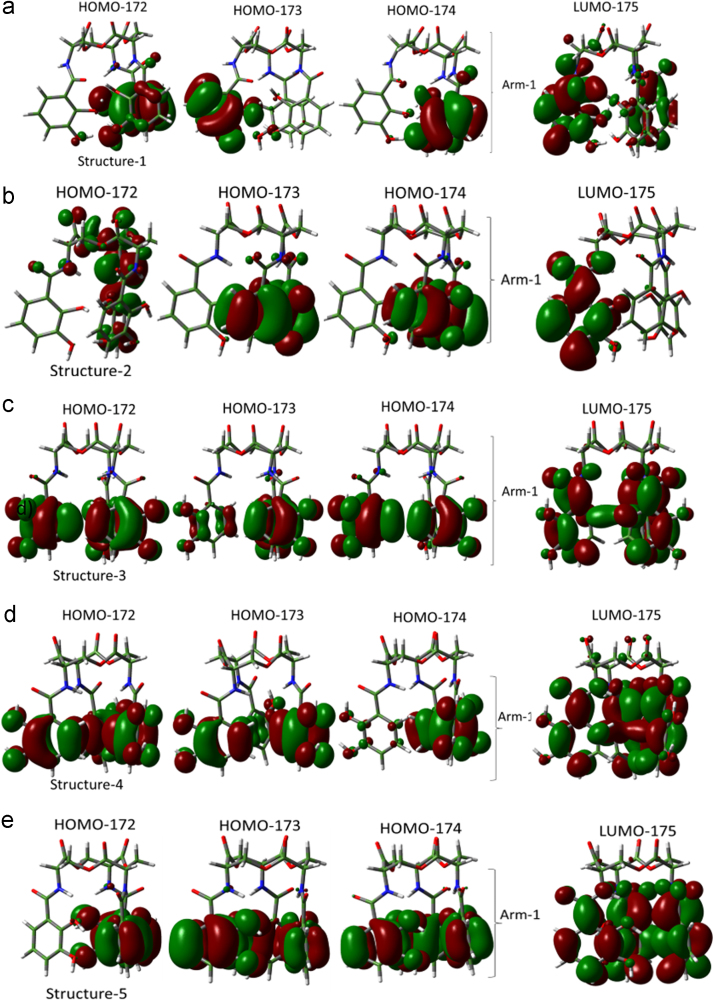
Frontier Orbitals (HOMO-LUMO) of structure 1(a), structure-2(b), structure-3(c), structure-4(d) and structure-5(e).

Unlike the H_6_EB [1], the calculated [CatFeEB]^3^^−^ shows a unique broad and sharp N-H band at 3547 cm^−^^1^, coherent with steric restrictions associated to the Fe, and as it is expected the stretching OH bands localized at 3812, 3846, 3747, 3522, 3420, 3371, 3221 and 2880 cm^−^^1^ in H_6_EB disappear in calculated [CatFeEB]^3^. Instead, this band is present in experimental FeEnterobactin, associated to the Fe linked in the Salicylate conformation [SalFeH_3_EB]^0^ as it is reported by N.K. Raymond [10], [11]. In the case of stretching and bending C-O bands its intensity decreases, and/or in some cases a signal shift is observed for 1336, 1235, 1175, 1125, 1032, 980, 849, 801, 695, 535 to 1378, 1361, 1094, 1064, 990, 943, 913, 857, 673, 629 and 544 cm^−^^1^ in [CatFeEB]^3^^−^, details of the H_6_EB IR can be found in [1]. [CatFeEB]^3^^−^ data revels signal shifts for the stretching (C=C) IR bands from 1587, 1544, 1468, 1390, 1343 to 1574, 1555, 1466, 1450, 1370 and 1335 cm^−^^1^, respectively, this is due to the inductive effect of the Fe attached to the catechol groups, similar to the reports from N.K. Raymond [10], [11]. The IR data is used as guide to improve the elucidation of FeEnterobactin and analogs. MALDI-TOF MS data of [CatFeEB]^3^^−^ exhibits a cleavage in C5-C4 instead C4-N in H_6_EB, again, it seems to be that the steric restrictions of the Fe linked to catechol leave the bond C5–C4 more reactive than C4-N in H_6_EB (see Fig. 2, Fig. 3). This is reflected in the dependence of frontier orbitals (HOMO-LUMO) with the frequency distribution of catecholamide dihedral angles of H_6_EB depicted in Fig. 4, Fig. 5, Fig. 6, Fig. 7, Fig. 8, for five H_6_EB structures. Despite of this wide versatility, the catecholamide arms tend to converge in only one range of frequencies; from − 60° to 60°, granting to H_6_EB a predominant reactive region governed for carbonyl groups (amide and ester). This match with the C4-N scission reveled from the MALDI-TOF MS data [1]. Fig. 8 depicts the highest occupied molecular orbital (HOMO) and lowest occupied molecular orbital (LUMO) of H_6_EB structures, where the effects of the dihedral angles are evident. They show an asymmetrical distribution of the ability to donate electrons (HOMO) and accept electrons (LUMO) located in the catecholamides arms.

Based in other analyzes by Vonlanthen et al. [13] and Mishchenko et al. [14] for a study of molecular conductance in a series of organic molecules with fixed dihedral angles, it is expected that the dihedral angles influence the properties of siderophores and their analogs as reported by Raymond et al. [15].

Thus, data here allow us to infer that the IR spectra and the reactivity are strongly influenced by the presence of Fe. These, together with the steric effects between the arms of catecholamide and with the trilactone backbone, as it is showed in data here. The reactive regions in [CatFeEB]^3^^−^ and H_6_EB, where the delocalization of electrons (amide, esters, and catechol groups) is predominant, are like a protein recognition code, giving rise to cellular memory. Nevertheless, this is beyond the scope of this contribution.

## 2 Experimental design, materials, and methods

Experimental infrared spectra were recorded at 50000 scans recorded with 2 cm^−1^ resolution. Samples, [SalFeH_3_EB]^0^ and H_6_EB, were measured using KRS-5 disc. Fifty milligrams of [SalFeH_3_EB]^0^ and H_6_EB, separately, was dispersed in 100 µl of dichloromethane, then one drop was placed on a KRS-5 disc to dry. Solid H_6_EB and [SalFeH_3_EB]^0^ were characterized. All solvents and H_6_EB were of analytical purity. For the sample preparation of MALDI-TOF MS spectra, 0.5 mL of a saturated solution of a-cyano-4-hydroxycinnamic acid (HCCA) in acetone was deposited on the sample target. A 1 ml aliquot of the sample was injected into a small drop of water previously deposited on the matrix surface.

Quantum Chemical calculations were performed using Density Functional Theory (DFT) with the PBE exchange-correlation functional including long-range corrections [6] and QZVP [7], [8] basis sets, with an ultrafine integral grid. Different starting catechol amide dihedral angles of H_6_EB were considered for the calculations (see data in Fig. 3, Fig. 4, Fig. 5, Fig. 6). All the results presented correspond to a local minimum for each of the calculated structures. All theoretical results were performed with the Gaussian 09 code [9] and we used Gauss-View to visualize the molecular orbitals, electrostatic potentials, and the vibrational modes. To obtain the frequencies of different dihedral angles values (Arm1, Arm2, and Arm3) from H_6_EB structures over a time lapse, molecular dynamics (MD) simulations (using the Desmond code) of the four structures of H_6_EB were performed, where each structure was embedded into an explicit TIP3P [2] water box. The NPT ensemble was employed with at 300 K and 1.01 bar of pressure and the OPLS-2005 force field [3] was used. Each system was subjected to energy minimization before the MD simulations were carried out for 5 ns. We used a VMD software [5] to calculate the dihedrals angles on catecholamides from H_6_EB structures during the MD trajectories. Plots were done with Origin 6.0 (OriginLab, Northampton, MA). All systems were simulated considering periodic boundary conditions (PBC).

## Footnotes

Transparency document Transparency data associated with this article can be found in the online version at https://doi.org/10.1016/j.dib.2018.08.114.
