# Supplementary material for: Experimental and theoretical structural/spectroscopical correlation of enterobactin and catecholamide
Source: Data Brief. 2018 Aug 29;20:2054–64. doi: 10.1016/j.dib.2018.08.114 (PMC6178210; doi:10.1016/j.dib.2018.08.114)
Supplement: Supplementary file 1 — Supplementary material [file mmc1.docx]

Conflict of Interest Form
